# Signaling networks controlling ID and E protein activity in T cell differentiation and function

**DOI:** 10.3389/fimmu.2022.964581

**Published:** 2022-08-02

**Authors:** Sung-Min Hwang, Sin-Hyeog Im, Dipayan Rudra

**Affiliations:** ^1^ Department of Obstetrics and Gynecology, Weill Cornell Medicine, New York, NY, United States; ^2^ Department of Life Sciences, Pohang University of Science and Technology, Pohang, South Korea; ^3^ Institute for Convergence Research and Education, Yonsei University, Seoul, South Korea; ^4^ ImmunoBiome Inc., Bio Open Innovation Center, Pohang, South Korea; ^5^ School of Life Science and Technology, ShanghaiTech University, Shanghai, China

**Keywords:** E proteins, ID proteins, E-ID axis, T cell differentiation, T cell function, regulatory T (Treg) cells, signaling pathways

## Abstract

E and inhibitor of DNA binding (ID) proteins are involved in various cellular developmental processes and effector activities in T cells. Recent findings indicate that E and ID proteins are not only responsible for regulating thymic T cell development but also modulate the differentiation, function, and fate of peripheral T cells in multiple immune compartments. Based on the well-established E and ID protein axis (E-ID axis), it has been recognized that ID proteins interfere with the dimerization of E proteins, thus restricting their transcriptional activities. Given this close molecular relationship, the extent of expression or stability of these two protein families can dynamically affect the expression of specific target genes involved in multiple aspects of T cell biology. Therefore, it is essential to understand the endogenous proteins or extrinsic signaling pathways that can influence the dynamics of the E-ID axis in a cell-specific and context-dependent manner. Here, we provide an overview of E and ID proteins and the functional outcomes of the E-ID axis in the activation and function of multiple peripheral T cell subsets, including effector and memory T cell populations. Further, we review the mechanisms by which endogenous proteins and signaling pathways alter the E-ID axis in various T cell subsets influencing T cell function and fate at steady-state and in pathological settings. A comprehensive understanding of the functions of E and ID proteins in T cell biology can be instrumental in T cell-specific targeting of the E-ID axis to develop novel therapeutic modalities in the context of autoimmunity and cancer.

## Introduction

A diverse network of transcription factors (TFs) and modulators regulate the expression of relevant genes involved in lymphocyte generation and function (1, 2). E and ID proteins are well-characterized transcriptional regulators that belong to the helix-loop-helix (HLH) family of proteins (3). They are widely recognized to play a significant role in developing lymphocytes, particularly B and T cells (4–9). E and ID proteins are crucially involved in various stages of thymic T cell development. For instance, E2A and HEB, which represents the major E proteins, have been demonstrated to play crucial roles in the early stages of thymocytes differentiation (10–13).

Several types of E proteins are identified, forming active homo- and heterodimers within the HLH proteins, binding to DNA, and regulating the transcription of multiple target genes in T cells (7, 8). Considerable evidence in mice shows that active E proteins are primarily engaged in the generation, differentiation, and effector function of different peripheral CD4 T cell subsets and CD8 T cell populations. On the other hand, ID proteins, encoded by four different genes (Id1-Id4), lack DNA binding activity and, through E-ID heterodimerization, modulate gene expression primarily by interfering with the DNA binding and transcription-related activities of E proteins (5). While various transcription factors regulate the expression of E proteins, the ID proteins are the only regulators that inhibit the transcription factor activity of E proteins through a mechanism that interferes with the formation of dimers within E proteins. Unlike E proteins, which are ubiquitously expressed in many tissues and cells, ID proteins are found to be expressed in a tissue- and cell-specific manner (14). For instance, Id2 and Id3 are predominantly expressed in T cells compared with Id1 and Id4. Indeed, Id2 and Id3 are recognized for their crucial roles in multiple discrete steps of T cell development and the differentiation and effector function of various CD4 and CD8 T cells. They have also been shown to suppress the generation of innate-like γδ (15) and invariant NKT (iNKT) cells (16), reinforcing αβ CD4 and CD8 T cell development in the thymus.

As E and ID proteins play critical roles in T cells through well-established molecular dynamics; the balance between these proteins has the potential to alter the E-ID axis-mediated global transcriptional program, affecting T cell phenotypes, and contributing to many aspects of autoimmune diseases, inflammation, and cancer progression (17). It is becoming clear that various TFs and extrinsic signaling pathways can affect the expression and stability of E and ID proteins by influencing the interactions between them. This review focuses on a brief overview of E and ID proteins and their molecular relationship, the interplay between E and ID proteins in the regulation of peripheral T cell activation, differentiation, and function, and the mechanisms by which TFs and extrinsic signaling pathways act on altering the E-ID axis in a context-dependent manner to dictate T cell function and fate.

## Molecular dissection of E and ID proteins

For decades, it has been well known that E and ID proteins interact closely with each other as transcription regulators, and their structure and mode of interaction are established. This section will describe the overview of E and ID proteins, highlighting the protein structure/domains and molecular features of their interaction in the context of transcription regulation.

### E proteins

**Figure 1:**
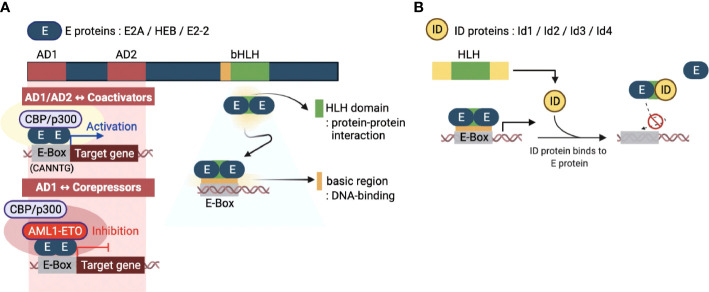
Molecular basis of E and ID protein functions. **(A)** An overview of the key domains of E proteins (E2A, HEB, and E2-2) - transcriptional activation domains (AD1 and AD2) and basic helix-loop-helix domain (bHLH). The AD1 and AD2 domains enable the E-box sequence (CANNTG) bound E protein homodimerization. E proteins function as transcriptional activators or repressors through recruitment of coactivators (CBP/p300) or corepressors (AML1-ETO, acute myeloid leukemia1-eight-twenty one oncoprotein), respectively. The bHLH domain consists of two parts: the HLH domain for protein-protein interactions and the basic region for DNA binding. **(B)** The four inhibitors of DNA binding (ID) proteins have one HLH domain in common. The ID protein interferes with the homo- or heterodimerized E proteins through this domain, inhibiting their DNA binding and transcription-related activities. This figure was created with BioRender.com.

E proteins are a family of TFs that recognize a consensus DNA sequence (CANNTG) known as an enhancer box (E-box). E proteins are encoded from three genes, E2A, HEB, and E2-2, which encode multiple proteins through alternative splicing. The E2A gene encodes E12 and E47 proteins, while the HEB and E2-2 genes encode both canonical (HEBcan and E2-2can) and alternative splice variants (HEBalt and E2-2alt) proteins (5). These E proteins are ubiquitously expressed and function in many tissues and cell types. E proteins contain several conserved domains, including the basic HLH (bHLH) domain and transcriptional activation domains (AD) (**Figure 1A**) (18). A C-terminal bHLH domain consists of approximately 60 amino acids and has two functionally distinct regions: the basic and HLH regions. The basic part is essential for initiating or repressing gene transcription by binding to the E box present downstream of specific target genes. On the other hand, the HLH region contains two amphipathic α-helices with a linking loop and is required for protein-protein interaction with other HLH proteins. Since the E proteins bind to the E-box of genomic DNA by forming homo- or heterodimers to initiate the transcription of target genes, the bHLH domain is an essential part of these two distinct processes governing the transcriptional machinery of the E proteins. In addition, E proteins also contain two transcriptional activation AD domains, AD1 and AD2. These two domains have been shown to recruit co-transcriptional activators, such as CBP/p300 and Spt/Ada/Gcn5 acetyltransferase (SAGA) complex (**Figure 1A**), to promote the transcriptional activity of a target gene (19). Contrary to acting on transcriptional activation, it has been demonstrated that corepressors, such as ETO family proteins and leukemogenic AML1-ETO fusion protein, can interact with AD1 (**Figure 1A**) (20, 21). These interactions contribute to transcriptional repression mechanisms of E proteins by inhibiting the recruitment of coactivators on target genes

### ID proteins

The inhibitor of DNA binding (ID) proteins, Id1-Id4, are also members of the HLH protein family. Regarding protein structure/domain and function, all four ID proteins have highly conserved common domains and similar molecular functions. Although ID proteins contain the same HLH domain as E proteins, homodimers or heterodimers between ID proteins can occur in rare circumstances (22). Interestingly, however, they can heterodimerize with bHLH proteins, primarily by inhibiting the formation of DNA-bound bHLH dimers (5). The lack of a basic region of the HLH domain distinguishes the ID proteins from the E proteins. Therefore, ID proteins cannot bind to the promoter regions and directly mediate the transcriptional regulation of genes. Thus, ID proteins operate as dominant-negative regulators of bHLH TFs and indirectly repress transcription of E protein target genes (**Figure 1B**). Interestingly, there is also evidence that ID proteins have functions unrelated to E proteins (23). The exact mechanism of such ‘non-canonical’ E-protein independent functions of ID proteins in transcriptional regulation is unknown and requires further investigation.

ID proteins are expressed in various tissues and cell types, including neuronal and immune compartments tumors (24–26). In particular, Id2 and Id3 are dominantly expressed in immune cells and have been demonstrated to control the expression of different genes that play critical roles in the development, differentiation, and function of T cells in steady-state and pathological conditions. In the following section, therefore, we will discuss the effects of ID proteins on the activation and function of T cells through their interaction with E proteins.

## The E-ID axis orchestrates peripheral T cell differentiation and function

T cells are one of the key immune cell types that comprise the adaptive immune system, offering cellular protection against pathogenic assaults and capable of eliciting a long-lasting memory response. T cells begin their life cycle as T cell precursors generated in the bone marrow and migrate to the thymus, where they undergo successive stages of development and maturation. During this process, thymic T cell precursors initiate genetically programmed transcriptional cascades that rely on precise functional networks of multiple TFs in response to thymus-related environmental signals (27). Following that, naive T cells matured in the thymus undergo further proliferation and activation as they migrate to secondary lymphoid organs. They are activated upon engagement with peptide-MHC on APC through TCR-CD3 complex molecules and CD28-mediated co-stimulation. In addition, the cytokine milieu act as an essential tertiary factor that determines the differentiation and effector function of specific CD4 T helper cell (Th) subsets, like Th1, Th2, Th17, induced regulatory T cells (iTreg), and T follicular helper T cells (Tfh) as well as promotes the function of cytotoxic CD8 T cells (28). During this process, T cells that receive these signals activate a network of multiple TFs. Numerous studies have revealed different molecular pathways with varied implications on T cell activation, differentiation, function, proliferation, survival, and memory formation (29). The E-ID axis is believed to be responsible for regulating the transcription of several genes involved in the development and function of T cells. In this section, we will discuss the role of the E-ID axis in determining the differentiation and function in different peripheral T cell subsets in steady-state and pathological conditions.

### Th1 and Tfh cells

**Figure 2:**
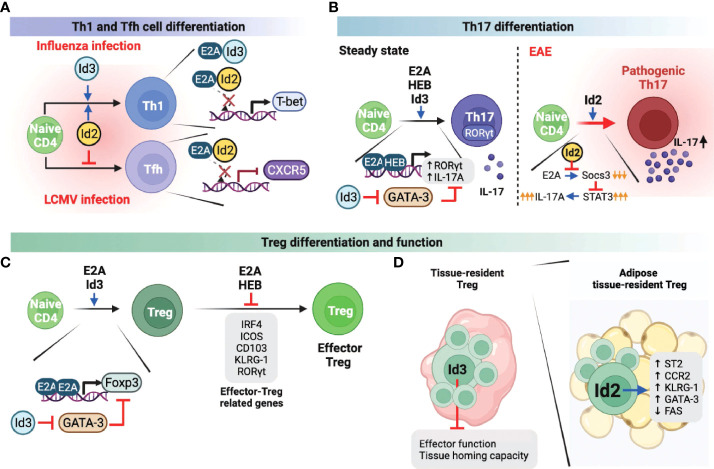
Role of E and ID proteins during peripheral CD4 T cell differentiation and function. A-C. E and ID proteins influence Th1 and Tfh cell differentiation **(A)**, Th17 cell differentiation and function **(B)**, and Treg and effector Treg differentiation and function **(C)** under steady-state as well as infectious and autoimmune conditions. **(D)** Id3 negatively influences the tissue-resident Treg effector function and tissue homing capacity. In adipose tissue, tissue-resident Treg cells enhanced Id2 expression, which can positively regulate the Treg effector function and survival. This figure was created with BioRender.com.

When a viral infection occurs, the immune system induces naive CD4 T cells to actively differentiate into two lineages: Th1 cells and Tfh cells (30, 31). Th1 cell differentiation drives inflammatory responses and pathogen clearance, whereas Tfh cells enhance germinal center (GC) responses for forming high-affinity antibodies and immunological memory against the virus. While Th1 and Tfh differentiation occur concurrently, these T cell identities are mutually exclusive and are governed by T-bet and Bcl6, which are the master regulators for the differentiation of Th1 and Tfh cells, respectively (32, 33). In the case of Th1 cells, it has been reported that both Id2 and Id3 promote Th1 differentiation in the context of influenza viruses by promoting the expression of T-bet, which is negatively regulated by the E proteins (34). In line with this finding, another study demonstrated that enhanced Id2 expression promotes Th1 differentiation while suppressing E protein-mediated CXCR5 expression, which is essential for Tfh cell differentiation and maturation upon lymphocytic choriomeningitis virus (LCMV) infection (**Figure 2A**) (35). Id2, therefore reciprocally modulates Th1/Tfh cell differentiation in the course of viral infection and promotes cell-mediated immunity, which does not rely on Tfh cell-mediated humoral response mechanisms to respond to virus infection.

### Th17 cells

Th17 cells are one of the CD4 T cell subsets that play a prominent role in maintaining mucosal barrier homeostasis by contributing to pathogen clearance at mucosal surfaces (36, 37). Loss of Th17 populations in the gut mucosal sites is directly associated with increased microbial translocation into the normal sterile tissues, leading to systemic immune activation and inflammation. However, excessive or uncontrolled Th17 activation has been linked to several autoimmune diseases, including multiple sclerosis (MS), arthritis, psoriasis, and lupus (38, 39). Therefore, it is important to understand the mechanisms involved in the differentiation and function of Th17 cells in both homeostatic and pathological settings. Not surprisingly, E and ID proteins are also involved in Th17 differentiation and function. For example, E2A and HEB were found to directly induce RORγt and interleukin-17 (IL-17) (**Figure 2B**), which are important for the differentiation and function of Th17 cells, respectively (40). Interestingly and somewhat counterintuitively, Id3-deficient naïve CD4 T cells exhibit decreased Th17 differentiation relative to wild-type T cells *in vitro*. Id3 deficiency leads to increased GATA-3 expression, which suppresses RORγt expression (40). In another study, Id2 was associated with increased activation phenotype of CD4 T cells under steady-state conditions, as well as IL17 production upon experimental autoimmune encephalomyelitis (EAE), an animal model of MS, induction, ultimately contributing to severe EAE pathogenesis (**Figure 2B**) (41). Mechanistically, increased Id2 in activated T cells suppresses E protein-mediated expression of Socs3. Socs3 is a negative regulator of cytokine production through the JAK/STAT pathway (42). Thus enhanced expression of Id2 results in restoration of IL-17A production that is otherwise suppressed by Socs3 (**Figure 2B**). Taken together, E and ID proteins promote Th17 cell differentiation and function through different regulatory mechanisms, respectively.

### Treg cells

The functions of E and ID proteins in Treg cells have also been extensively studied and led to complicated conclusions. Unlike other T cell subsets, Treg cells are a unique subset of CD4 T cells, indispensable for peripheral tolerance (43). It was reported that E2A directly promotes the expression of Foxp3, a well-defined Treg lineage specificity factor, by binding at the *Foxp3* promoter (**Figure 2C**) (44). In addition, Id3 suppressed GATA-3 expression, which represses the transcription of *Foxp3*, suggesting that E and ID proteins contribute independently of each other towards optimal Foxp3 expression (**Figure 2C**). In another study, however, E2A and HEB were found to negatively regulate Foxp3 and other effector-related factors in Treg cells such as IRF4, ICOS, CD103, KLRG-1, and RORγt, and consequently inhibited effector Treg differentiation and function (**Figure 2C**) (45). Based on the conflicting results of E proteins regulating *Foxp3* transcription, the mechanisms by which E proteins regulate *Foxp3* appear to differ, depending on the effector stages of Treg cells, and therefore require further investigation. On the other hand, recent studies discovered that ID proteins play an important role in the differentiation, function, and survival of tissue-resident Treg cells (**Figure 2D**). For instance, Id2 is highly expressed in adipose-resident Treg cells and is associated with increased expression of the adipose Treg-related genes *Il1rl1* (codes for the IL33 receptor ST2), *Ccr2*, *Klrg1*, and *Gata3*, but suppresses the apoptosis-related gene, *Fas* (46). Another group demonstrated that Id3 is directly associated with decreased effector function and tissue homing capacity of tissue-resident Treg cells (47). However, establishing a direct role of Id3 downregulation in the functional differentiation of tissue-resident Treg cells requires further investigation. ID protein-mediated Treg differentiation and function also contribute to the pathogenicity of autoimmune diseases. In systemic lupus erythematosus (SLE) patients, Id3 expression levels were positively correlated with Treg cell frequencies, and subsequently, mice in which Id proteins were overexpressed showed favorable autoimmune responses (48). In contrast, elevated Id3 expression was found to promote Treg differentiation in hepatitis B virus infection, thereby reducing viral clearance and developing a chronic state of infection (49). Thus, these two independent studies demonstrated that disease prognosis might differ based on Treg differentiation and function, suggesting the importance of targeting Id3 expression in Treg cells according to its context.

### CD8 T cells

**Figure 3:**
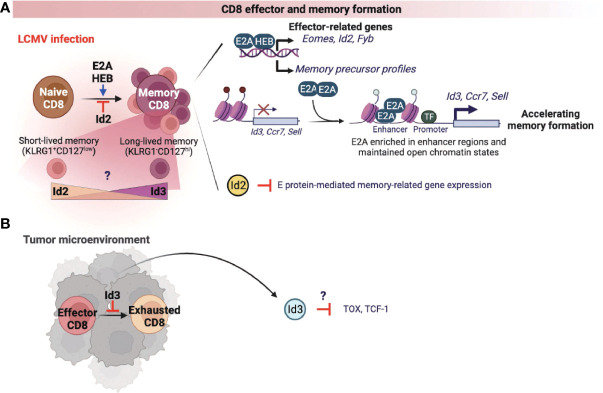
E-ID axis in the formation of effector and memory CD8 T cells. E and ID proteins regulate effector and memory CD8 T cell differentiation and function in infection **(A)** and cancer **(B)**. This figure was created with BioRender.com.

CD8 cytotoxic T cells are well known for anti-viral immune responses and anti-tumor immunity (50). Currently, there are two studies on the role of the E proteins in association with the formation of memory CD8 T cells responding to infection. One study demonstrated that both E2A and HEB transcriptionally upregulate the effector-associated genes such as *Eomes*, *Id2*, and *Fyb* and increase the generation of memory precursor T cells (51). The other study showed that E2A epigenetically regulates the accessibility of enhancers of memory-related genes such as *Id3*, *Ccr7*, and *Sell*, increasing the frequency of memory precursor effector cells and accelerating memory cell formation (**Figure 3A**) (52). As expected, Id2 expression suppressed E proteins-mediated gene expression, thereby suppressing the differentiation of memory CD8 T cells (**Figure 3A**) (53–55). Memory T cells are classified into two types according to the classification of Killer Cell Lectin Like Receptor G1 (KLRG1) and CD127. First, KLRG1^+^CD127^low^ cells are classified as short-lived effector memory cells, and most of them show rapid effector function and die. Conversely, KLRG1^-^CD127^hi^ long-lived memory T cells present a vital protective role during acute rechallenge with pathogens such as viruses or bacteria. Interestingly, in the context of LCMV infection, Id2 expression promoted the differentiation of short-lived effector-memory CD8 T cells, while Id3 expression demonstrated functional capability to induce differentiation of long-lived memory progenitors (**Figure 3A**) (56). However, the exact underlying mechanisms by which Id2 and Id3 are involved in the differentiation process of each memory cell types are yet to be elucidated. Therefore, it may be beneficial to investigate further how the expression of Id2 and Id3 is regulated in association with the signaling pathways that determine each memory T cell subset.

A recent study found that Id3 inhibits the exhaustion of CD8 T cells in the tumor microenvironment (TME) (**Figure 3B**) (57). Further, an elevated population of exhausted CD8 T cells was correlated with reduced anti-tumor immune response in TME. In fact, these exhausted CD8 T cells display high levels of Tcf1 and Tox, which are known representative markers of CD8 T cell exhaustion, and overexpression of these two factors also directly induces T cell dysfunction. These data warrants further investigations to understand whether Id3 can regulate Tcf1 or Tox expression (**Figure 3B**), which is implicated in the differentiation and function of exhausted CD8 T cells in the TME.

## Endogenous factors and cell-extrinsic signaling pathways impact the E-ID axis in T cell fate and function

**Figure 4:**
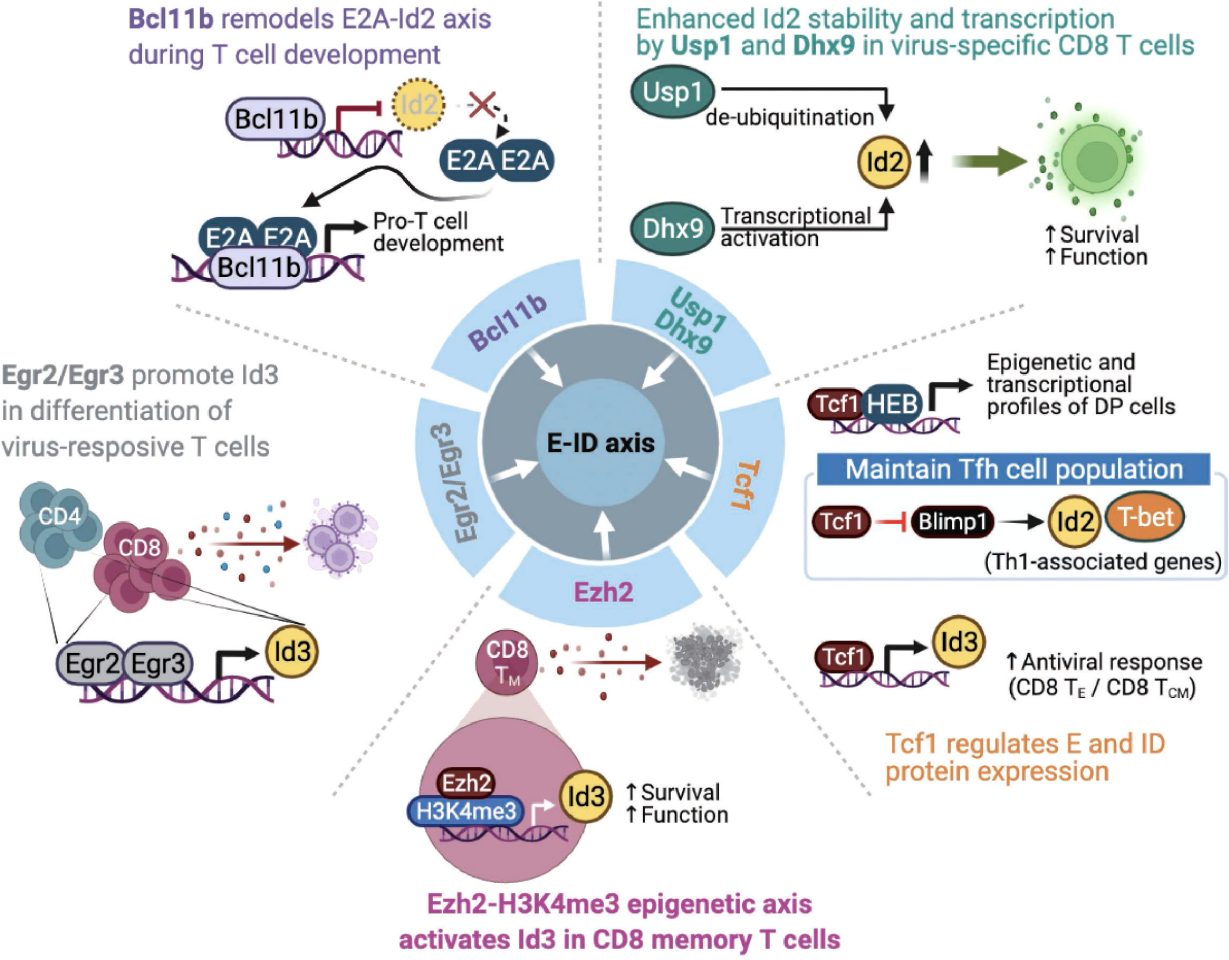
Endogenous factors influence the E-ID axis in T cell fate and function. The roles and mode of action of endogenous proteins that control E and ID protein expression and affect T cell development, differentiation, and function (Bcl11b, B-cell lymphoma/leukemia 11B; Egr2/Egr3, early growth response 2/early growth response 3; Ezh2, Enhancer of zeste homolog 2; H3K4me3, histone H3 lysine 4 trimethylation; Tcf1, T cell factor 1; Blimp1, B-lymphocyte-induced maturation protein 1; Usp1, ubiquitin-specific protease 1; Dhx9, DExH-Box Helicase 9; T_E_, Effector T cells; T_CM,_ Central Memory T cells). This figure was created with BioRender.com.

Recent studies have demonstrated that several endogenous proteins and extrinsic signaling pathways affect development, differentiation, function, and memory formation by altering the E-ID axis under steady-state and pathological conditions. In this section, we will emphasize some of the major endogenous proteins and extrinsic signaling pathways that influence the balance of the E-ID axis by controlling the expression of each of the E and ID proteins and their protein-protein interactions, leading to phenotypic changes and functional reprogramming of T cells in a context-dependent manner (**Figure 4**).

### Endogenous proteins associated with the E-ID axis

The zinc-finger transcription factor Bcl11b is a critical regulator of differentiation and survival during T cell development in the thymus (58–60). Hosokawa et al. discovered that Bcl11b, highly expressed at a specific pro-T cell lineage commitment stage (DN2-DN3), inhibits Id2 expression (61). In addition, the effect of depleting Bcl11b on gene expression in pro-T cells remarkably overlaps the impact of depleting E2A, suggesting that Bcl11b and E2A interact very closely during the pro-T cell development. In agreement with this finding, another study demonstrated that Bcl11b-dependent target genes are parallelly regulated by E2A during T cell development (62), indicating that Bcl11b plays a critical role in remodeling the E-ID axis through active suppression of Id2 expression. Given the importance of the E-ID axis in T cell development, further studies are needed to identify other transcription factors that contribute to the functionalities of the E-ID axis by regulating E and ID protein expressions.

Two other zinc finger transcription factors, Egr2 and Egr3, mediate self-tolerance by T lymphocytes and NKT cell development (63, 64). Miao et al. reported that Egr2 and Egr3 regulate clonal expansion and differentiation of virus-responsive T cells by directly promoting Id3 expression and other effector genes (65). Nevertheless, the exact mechanism remains unclear.

T cell factor 1 (Tcf1) is the key transcription factor of the canonical Wnt signaling pathway (66). Tcf1 plays an essential role in controlling T cell development, the differentiation of specific CD4 T helper (Th) subsets, and the formation of memory and stem-cell-like CD8 T cells following various types of viral infections (67). Tcf1 closely interacts with the E-protein HEB to establish epigenetic and transcriptional profiles of double-positive thymocytes (68). Mechanistically, TCF-1 inhibits Notch signaling, which protects HEB from Notch-induced proteasomal degradation, suggesting that Tcf1 is involved in the stability of E proteins. In response to acute viral infection, another study found that Tcf1 maintains T follicular T helper (Tfh) cell population by suppressing Blimp1, which promotes Th1-associated effector genes such as T-bet and Id2 expression in Tfh cells (69). Since E protein induces CXCR5, associated with Tfh cell migration into B cell follicles and subsequent further differentiation of Tfh cells (70), Tcf1-mediated Blimp1 repression may serve as a unique mechanism for maintaining the Tfh population in the context of viral infection. For CD8 T cells, a recent study discovered that Tcf1 directly modulates the expression of Id3, which is important for both effector and central memory function of CD8 T cells, thereby affecting optimal CD8 T cell activity in the context of viral infection (71). Further, ectopic expression of Tcf1 was associated with increased expression of Id3 and several key effector components known to counteract CD8 T cell exhaustion upon LCMV infection, eventually leading to reinforced CD8 T cells mediated antiviral response (72).

The histone methyltransferase Ezh2 is involved in forming CD8 T cell memory precursors and contributes to the antitumor activity of CD8 memory T cells in the tumor microenvironment. Ezh2 was found to promote the expression of Id3 for maintaining the function of effector and memory CD8 T cells. Interestingly, Ezh2 was found to promote the expression of Id3 by enhancing H3K4me3 modification on its gene locus, which is distinct from the well-known repressive H3K27me3 promoting activity of Ezh2 (73). Considering these exciting observations, further studies are required to clarify the precise roles of various epigenetic modifying enzymes related to the E-ID axis in CD8 memory T cell formation and function in tumor microenvironments.

Interestingly, recent studies have demonstrated that the E-ID axis involved in T cell function is also regulated by factors whose functions extend beyond transcription regulation. For example, the de-ubiquitinase Usp1, which is known to stabilize Id1-Id3, contributes to maintaining stem cell properties in osteosarcoma and mesenchymal stem cells (74, 75). In activated T cells, Usp1 interacts with Id2 and Id3, protects the stability of Id2 protein in the context of viral infection, and maintains the proliferative potential and memory phenotype differentiation of virus-specific CD8 effector T cells (76). Similarly, Jiao et al. demonstrated that DExD/H-box helicase 9 (Dhx9) is required for a proper CD8 T cell response against acute viral infection. Interestingly, contrary to the well-established role of Dhx9 as a cytosolic DNA-sensor, Dhx9 was found to directly increase the transcription level of Id2 expression, thereby affecting the survival and function of viral-specific CD8 T cells (77). Mechanistically, the authors discovered that two domains of Dhx9, double-stranded RNA binding motif (DSRM) and oligonucleotide/oligosaccharide binding fold (OB_Fold) domain, play an essential role in directly binding to the *Id2* promoter and consequently regulating the level of Id2 transcription.

### Extrinsic signaling pathways associated with the E-ID axis

**Figure 5:**
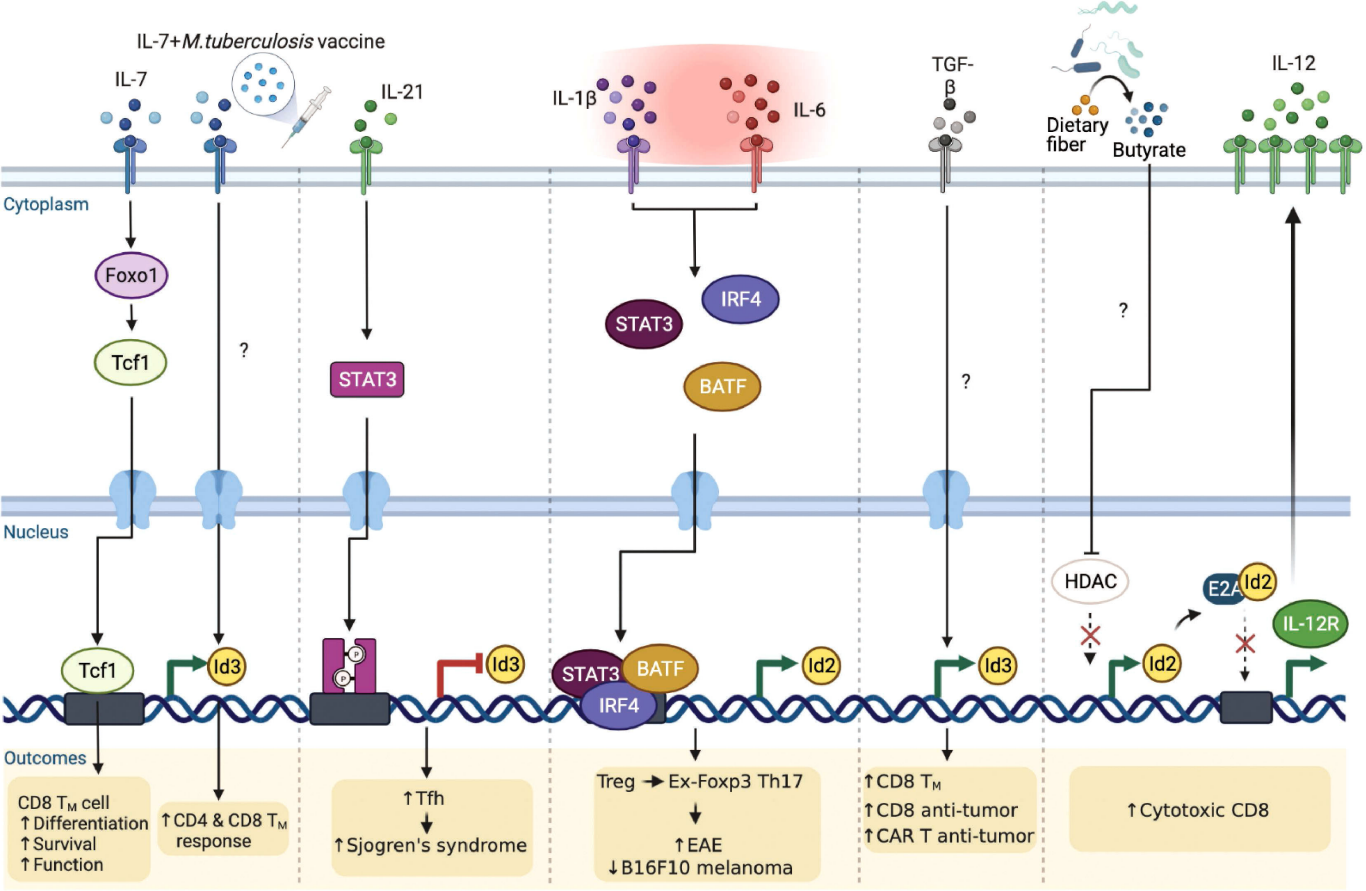
Extrinsic signaling pathways affect E-ID axis-mediated T cell function in infection, autoimmunity, and cancer. External signaling pathways affect the expression of E and ID proteins as well as the E-ID axis in various T cell subsets, resulting in changes in the phenotype of T cells and alleviating or exacerbating diseases (Foxo1, Forkhead Box O1; STAT3, Signal Transducer And Activator Of Transcription 3; IRF4, Interferon Regulatory Factor 4; BATF, Basic Leucine Zipper ATF-Like Transcription Factor; TGF-β, Transforming growth factor β; HDAC, Histone deacetylase; T_M_, Memory T cells; CAR, Chimeric Antigen Receptor). This figure was created with BioRender.com.

It is becoming clear that extracellular cytokine signaling can further modulate the E-ID axis dependent transcriptional reprograms in a T cell lineage-specific and context-dependent manner, consequently governing T cell phenotypes implicated in several disease outcomes (**Figure 5**). Interleukin-7 (IL-7) appears to be an important determinant in this context. A recent study discovered that Interleukin-7 (IL-7) signaling promotes Foxo1-Tcf1-Id3 pathways to maintain memory CD8 T cell differentiation, survival, and function (78). Although IL-7 signaling is well-established to play an important role in T cell survival and proliferation (79, 80), before this finding, it was primarily believed to function by enhancing the expression of anti-apoptotic Bcl-2 family of proteins, especially Mcl1 and Bcl-2 (81). In another study, Han et al. demonstrated that when mice were administered with the *Mycobacterium tuberculosis* (*M. tuberculosis*) subunit vaccine and adeno-associated virus-mediated IL-7, Id3 expression was directly upregulated, contributing to long-term memory CD4 and CD8 T cells response against *M. tuberculosis* infection (82). The underlying mechanism, however, is yet to be elucidated.

Interleukin-21 (IL-21) is an IL-2 family cytokine produced by activated T cells, mainly by natural killer T (NKT) cells, Th17 cells, and Tfh cells, to regulate immune responses (83, 84). Elevated amounts of IL-21 have been reported in several autoimmune diseases (85) such as inflammatory bowel disease (86), rheumatoid arthritis (87), type 1 diabetes (88), and systemic lupus erythematosus (89). Interestingly, a recent study demonstrated that IL-21 signaling directly inhibits Id3 *via* STAT3, promoting differentiation of hyper-activating Tfh cells, exacerbating the pathogenesis of Sjogren’s syndrome (90). Hence, it is evident that IL-21 affects the E-ID axis under specific inflammatory conditions, thereby influencing the differentiation and function of disease-related target cells. In addition to IL-21, Interleukin-2 (IL-2) and Interleukin-15 (IL-15) are also major cytokines that regulate T cell differentiation, proliferation, effector function, and memory formation (91, 92). Given that the E-ID axis is critical for effector T cell function and memory T cell formation, further investigation is required to determine how IL-2 and IL-15 downstream signaling affect the E-ID axis in this context.

Under the inflammatory milieu in the context of autoimmune diseases, Treg cells are known to convert into IL-17 producing cells (93–95). These Treg cells that have lost Foxp3 and become Th17 cells are called “ex-Foxp3 Th17” cells. Previously, our group demonstrated a unique mechanism by which the pro-inflammatory cytokines, IL-1β and IL-6, by altering the E-ID axis, contribute to Treg cell plasticity (96). In this context, we found that Id2 expression is intrinsically repressed during Treg cell differentiation, which is likely one of the reasons why Treg cells stably maintain the expression of Foxp3 in an E2A-dependent manner. In an inflammatory setting, particularly in experimental autoimmune encephalomyelitis (EAE), elevated IL-1β and IL-6 mediated STAT3/IRF4/BATF signaling cascades promote Id2 activation. Enhanced Id2 blocks the binding of E2A to the *Foxp3* locus and renders Treg cells into pathogenic ex-Foxp3 Th17 cells, resulting in exacerbated EAE pathogenesis. Interestingly, in mice bearing B16-F10 melanoma, artificially promoting the plasticity of Treg cells upon transient ectopic expression of Id2 effectively inhibited tumor growth (96). Thus, re-balancing the E-ID axis in a context-dependent manner may be beneficial in treating autoimmunity and cancer.

In terms of promoting the anti-tumor activity of tumor-infiltrating T cells in association with the E-ID axis, a recent study showed that *ex-vivo* stimulation of human T cells with exogenous transforming growth factor beta (TGFβ) leads to the accumulation of central memory T cells that exhibit relatively superior antitumor function than effector T cells (97). This is achieved by upregulating the memory-associated regulatory factor Id3 and improving the anti-tumor activity of T cells and chimeric antigen receptor-expressing T cells. However, the role played by TGFβ leading to the upregulation of Id3 and the target genes regulated downstream of the E-ID axis in this context requires further investigation.

Moreover, it is worth mentioning that metabolites derived from intestinal microbes can directly regulate the E-ID axis, affecting anti-tumor immunity. A recent study demonstrated butyrate, one of the short-chain fatty acids (SCFAs), to be directly involved in the upregulation of Id2 expression by inhibiting histone deacetylase (HDAC) activity (98). Elevated Id2, interfering with the activity of E2A, restores the expression of E2A-repressed IL-12R and consequently activates IL-12 signaling, which is important for the cytotoxic activity of CD8 T cells. Curiously enough, this Id2-expression promoting activity of butyrate appears to be a cell type and microenvironment-specific phenomenon since, contrary to CD8 T cells, butyrate is known to promote iTreg induction from TCR-stimulated CD4 T cells in the presence of TGFβ (99, 100), which according to our finding is negatively affected by enhanced expression of Id2. Thus, it will be interesting to determine further how specific metabolites of gut microbiota associated with cancer and inflammatory diseases affect T cell differentiation and function within the E-ID axis.

## Concluding remarks

In the past few years, several studies have clarified the functions and mechanisms of the E-ID axis determining T cell phenotypes in inflammation and cancer; however, further research is necessary to understand the role of E and ID proteins in the metabolic reprogramming of T cells in health and disease. In particular, an integrated mechanistic understanding of the E-ID axis regulating metabolic pathways or rate-limiting enzymes in glycolysis or mitochondrial fatty acid oxidation could provide opportunities for developing effective therapeutic interventions to promote the anti-cancer function of tumor-infiltrating T cells. Moreover, interesting areas focused on improving tumor immunotherapy are the reversal of T cell exhaustion and the maintenance of stem-like memory T cells, which have been demonstrated as long-lived, self-renewing T cell populations important for sustained antitumor immunity in the TME (101). The ID proteins not only inhibit the differentiation of exhausted CD8 T cells in the TME but are also involved in generating diverse memory CD8 T cell subsets under viral infection conditions. Therefore, understanding the E-ID axis in this context could contribute to developing new approaches to improving the better outcomes of cancer immunotherapy.

Besides T cells, several studies have suggested that ID proteins in cancer cells play an important role in promoting tumor progression and metastasis (14, 102). There is increasing evidence that the functional inhibition of ID proteins by pharmacological drugs in cancer cells suppresses cancer cell proliferation under physiological conditions. For example, a recent study showed that the chemical compound, AK-778-XXMU, is a potent Id2 antagonist that can be used to treat gliomas (14, 103). On the contrary, ID protein expression improves the function of tumor-infiltrating T cells and virus-reactive T cells. In line with this finding, further preclinical studies need to be conducted utilizing humanized immunocompetent mouse models to determine durable responses of the pharmacological agonists in promoting and stabilizing ID protein expression in T cells that may prevent disease progression and/or recurrence in patients.

In this review, we discussed the critical roles of the E-ID axis in controlling T cell homeostasis and function under steady-state conditions and various pathological settings. Understanding the complexities of the multiple factors and extrinsic signaling pathways associated with the E-ID axis and further defining the pros and cons of targeting the E-ID axis to affect T cell responses is likely to emerge as an area of enormous therapeutic relevance in the future for immune-mediated diseases and cancer.

## Author contributions

S-MH: conceptualization, investigation, writing, and visualization of the manuscript. S-HI and DR: conceptualization, investigation, review, manuscript editing, and funding acquisition. All authors contributed to the article and approved the submitted version.

## Funding

S-MH has been supported by AACR-Bristol Myers Squibb Immuno-Oncology Research Fellowship. DR is funded by a startup fund from the School of Life Science and Technology (SLST), Shanghaitech University, Shanghai, China.

## Conflict of interest

S-HI is the CEO of the company ImmunoBiome Inc.

The remaining authors declare that the research was conducted in the absence of any commercial or financial relationships that could be construed as a potential conflict of interest.

## Publisher’s note

All claims expressed in this article are solely those of the authors and do not necessarily represent those of their affiliated organizations, or those of the publisher, the editors and the reviewers. Any product that may be evaluated in this article, or claim that may be made by its manufacturer, is not guaranteed or endorsed by the publisher.
